# Supplementary material for: The interactome of intact mitochondria by cross-linking mass spectrometry provides evidence for coexisting respiratory supercomplexes
Source: Mol Cell Proteomics. 2017 Dec 8;17(2):216–32. doi: 10.1074/mcp.RA117.000470 (PMC5795388; doi:10.1074/mcp.RA117.000470)
Supplement: Supplemental Data [file supp_RA117.000470_133922_0_supp_23978_fzffwf.zip › spectra_annotation/mito_spectra_annotation/202-1-3-1-5-1.pdf]

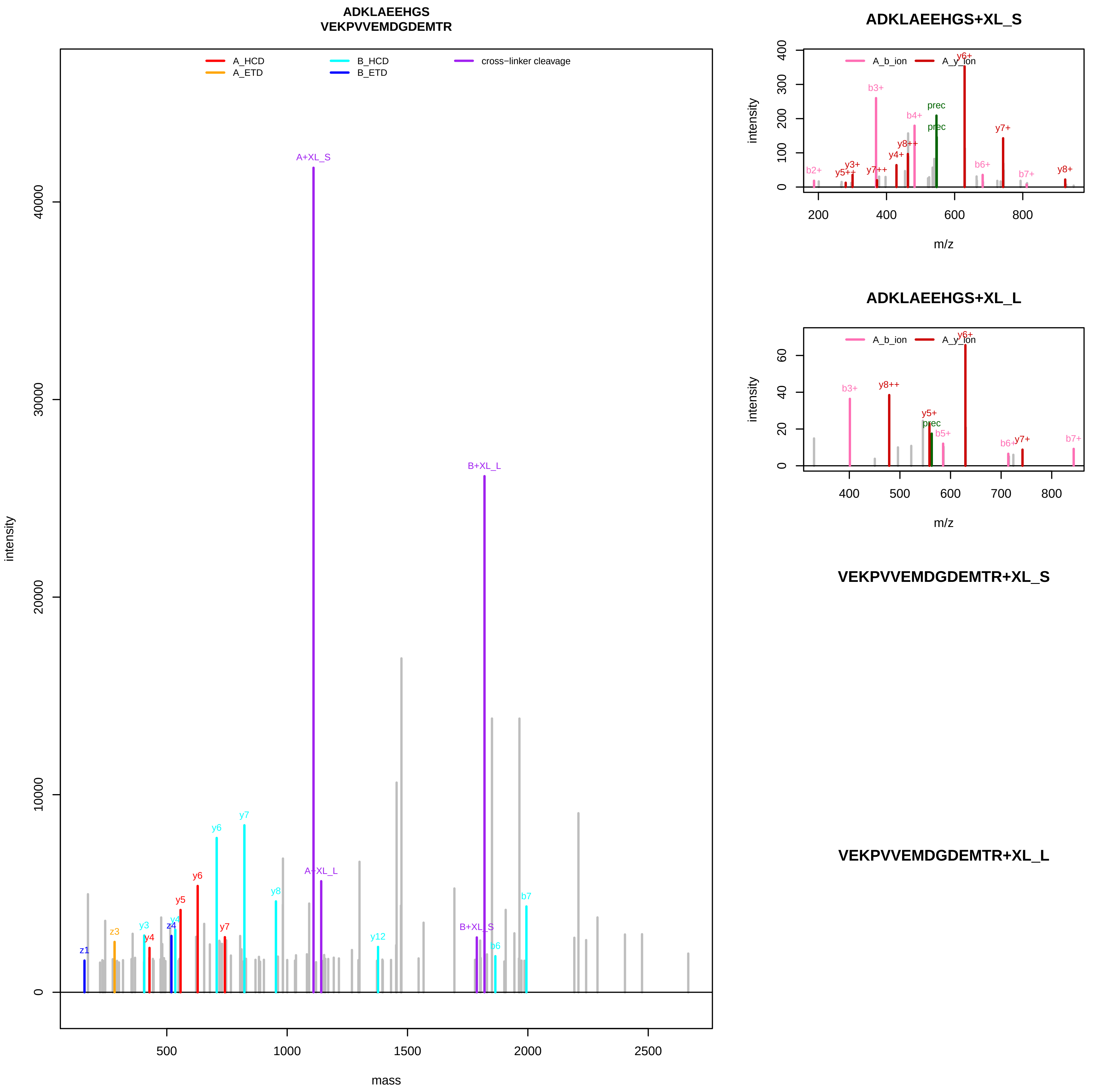
ADKLAEEHGS
VEKPVVEMDGDEMTR
A_HCD
A_ETD
B_HCD
B_ETD
cross−linker cleavage
A+XL_S
B+XL_L
A+XL_L
B+XL_S
z1
z3
y3
y4
z4
y4
y5
y6
y6
y7
y7
y8
y12
b6
b7
intensity
mass
0
10000
20000
30000
40000
500
1000
1500
2000
2500
ADKLAEEHGS+XL_S
A_b_ion
A_y_ion
b2+
y5++
y3+
y7++
b3+
y4+
y8++
b4+
prec
prec
y6+
b6+
y7+
b7+
y8+
intensity
m/z
0
100
200
300
400
200
400
600
800
ADKLAEEHGS+XL_L
A_b_ion
A_y_ion
b3+
y8++
y5+
prec
b5+
y6+
b6+
y7+
b7+
intensity
m/z
0
20
40
60
400
500
600
700
800
VEKPVVEMDGDEMTR+XL_S
VEKPVVEMDGDEMTR+XL_L
